# Evaluation of *Adenanthera pavonina*-derived compounds against diabetes mellitus: insight into the phytochemical analysis and *in silico* assays

**DOI:** 10.3389/fmolb.2023.1278701

**Published:** 2024-03-27

**Authors:** Md. Sojiur Rahman, Md. Eram Hosen, Md. Omar Faruqe, Md. Khalekuzzaman, Md. Asadul Islam, Uzzal Kumar Acharjee, Yousef A. Bin Jardan, Hiba-Allah Nafidi, Amare Bitew Mekonnen, Mohammed Bourhia, Rashed Zaman

**Affiliations:** ^1^ Professor Joarder DNA and Chromosome Research Laboratory, Department of Genetic Engineering and Biotechnology, University of Rajshahi, Rajshahi, Bangladesh; ^2^ Department of Computer Science and Engineering, University of Rajshahi, Rajshahi, Bangladesh; ^3^ Department of Pharmaceutics, College of Pharmacy, King Saud University, Riyadh, Saudi Arabia; ^4^ Department of Food Science, Faculty of Agricultural and Food Sciences, Laval University, Quebec City, QC, Canada; ^5^ Department of Biology, Bahir Dar University, Bahir Dar, Ethiopia; ^6^ Laboratory of Biotechnology and Natural Resources Valorization, Faculty of Sciences, Ibn Zohr University, Agadir, Morocco

**Keywords:** *Adenanthera pavonina*, phytochemicals, diabetes mellitus, 5hhw, molecular docking, molecular dynamics

## Abstract

*Adenanthera pavonina* is a medicinal plant with numerous potential secondary metabolites showing a significant level of antidiabetic activity. The objective of the current study was to identify potential phytochemicals from the methanolic leaf extract of *Adenanthera pavonina* as therapeutic agents against diabetes mellitus using GC-MS and *in silico* methods. The GC-MS analysis of the leaf extract revealed a total of 17 phytochemicals. Molecular docking was performed using these phytochemicals, targeting the mutated insulin receptor tyrosine kinase (5hhw), which inhibits glucose uptake by cells. Diazoprogesterone (−9.2 kcal/mol), 2,4,4,7a-Tetramethyl-1-(3-oxobutyl)octahydro-1H-indene-2-carboxylic acid (−6.9 kcal/mol), and 2-Naphthalenemethanol, decahydro-.alpha.,.alpha.,4a-trimethyl-8-methylene-, [2R-(2.alpha.,4a.alpha.,8a.beta.)] (−6.6 kcal/mol) exhibited better binding with the target protein. The ADMET analysis was performed for the top three compounds with the best docking scores, which showed positive results with no observed toxicity in the AMES test. Furthermore, the molecular dynamics study confirmed the favorable binding of Diazoprogesterone, 2,4,4,7a-Tetramethyl-1-(3-oxobutyl)octahydro-1H-indene-2-carboxylic acid and 2-Naphthalenemethanol, decahydro-.alpha.,.alpha.,4a-trimethyl-8-methylene-, [2R-(2.alpha.,4a.alpha.,8a.beta.)] with the receptor throughout the 100 ns simulation period.

## Graphical Abstract

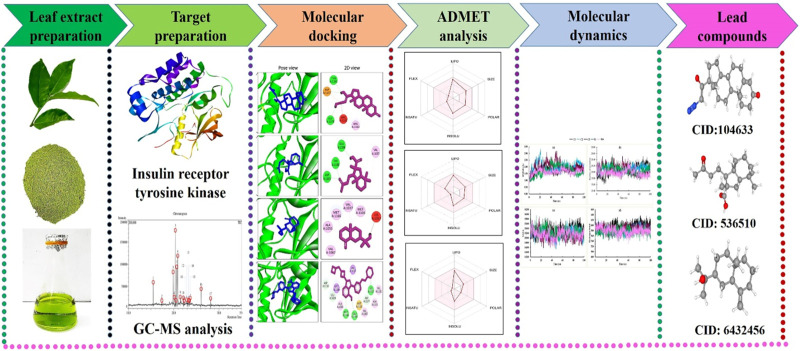

## 1 Introduction

*Adenanthera pavonina*, a member of the Fabaceae family, is a highly valued plant in traditional medicine due to its diverse medicinal properties. This plant species is known to be effective in treating various diseases, such as diabetes, lipid disorders, and diarrhea (Pandhare et al., 2012). The therapeutic potential of *Adenanthera pavonina* is attributed to its rich composition of phytochemicals, which includes steroids, glycosides, alkaloids, saponins, and polysaccharides (Wickramaratne et al., 2016). The plant extracts have been shown to exhibit a wide range of biological activities, including antidiabetic, antioxidant, anti-inflammatory and antimicrobial properties. Research on mice models has shown that *A. pavonina* leaf extract can effectively reduce the prevalence of diabetes mellitus (Wickramaratne et al., 2016).

Diabetes mellitus is one of the fastest-growing chronic metabolic disorders characterized by hyperglycemia (Karadag et al., 2018). In Bangladesh, India, and China, the number of patients suffering from diabetes mellitus type 2 (DMT2) has drastically increased (Goyal et al., 2020). Worldwide fast nutritional transitions, which involve altered eating habits, poor nutrition in early life and overnutrition in later life, can contribute to the DMT2 epidemic. In addition, Asians typically have a higher percentage of body fat mass, more abdominal obesity, and less muscle mass (Hills et al., 2018), which could account for their elevated susceptibility to DMT2. It has been also found that men are more prone to DMT2 than women (Iglay et al., 2016).

Insulin resistance and less insulin production are two key factors responsible for DMT2, which is characterized by abnormal metabolism of carbohydrates, proteins, and lipids by peripheral tissues (Saxena et al., 2019). DMT2 is far more prevalent than diabetes mellitus type 1 (DMT1). The primary cause of DMT2 is the increasing impairment in the ability of pancreatic beta-cells to secrete insulin, which typically occurs due to pre-existing insulin resistance in adipose tissue, liver, and skeletal muscle (Masini et al., 2017).

The insulin receptor (IR) is a tetrameric protein composed of two extracellular alpha and two transmembrane beta subunits (Hall et al., 2020). The tyrosine kinase beta subunit is activated through conformational changes induced by the binding of insulin to the alpha subunit of IR. Moreover, activated IR can phosphorylate and auto-phosphorylate intracellular substrates, both of which are necessary for initiating cellular responses to insulin (Haeusler et al., 2018). These actions activate downstream signaling molecules involved in the insulin-signaling pathway (Brierley et al., 2018). Impaired insulin signaling, including mutations in the insulin receptor tyrosine kinase (5hhw), is present in most diabetes mellitus patients. This insulin resistance contributes to hyperglycemia and other metabolic disorder (Nolan et al., 2019). Therefore, drugs that enhance insulin receptor tyrosine kinase activity might be helpful to deal with DMT2.

There are several medications to treat diabetes mellitus, such as insulin and metformin, which are costly and responsible for different types of side effects in the human body (Bao et al., 2021). However, natural plant-derived phytochemicals are relatively safe and less expensive. *A. pavonina* has been reported to possess bioactive substances that fight against DMT2 (Pandhare et al., 2012). Therefore, the information gathered from the molecular docking investigation of substances from *A. pavonina* with the 5hhw protein is crucial. The present study was conducted to identify potential activators of 5hhw and to report the interactions of phytochemicals with the target protein for further investigation.

## 2 Materials and methods

**FIGURE 1:**
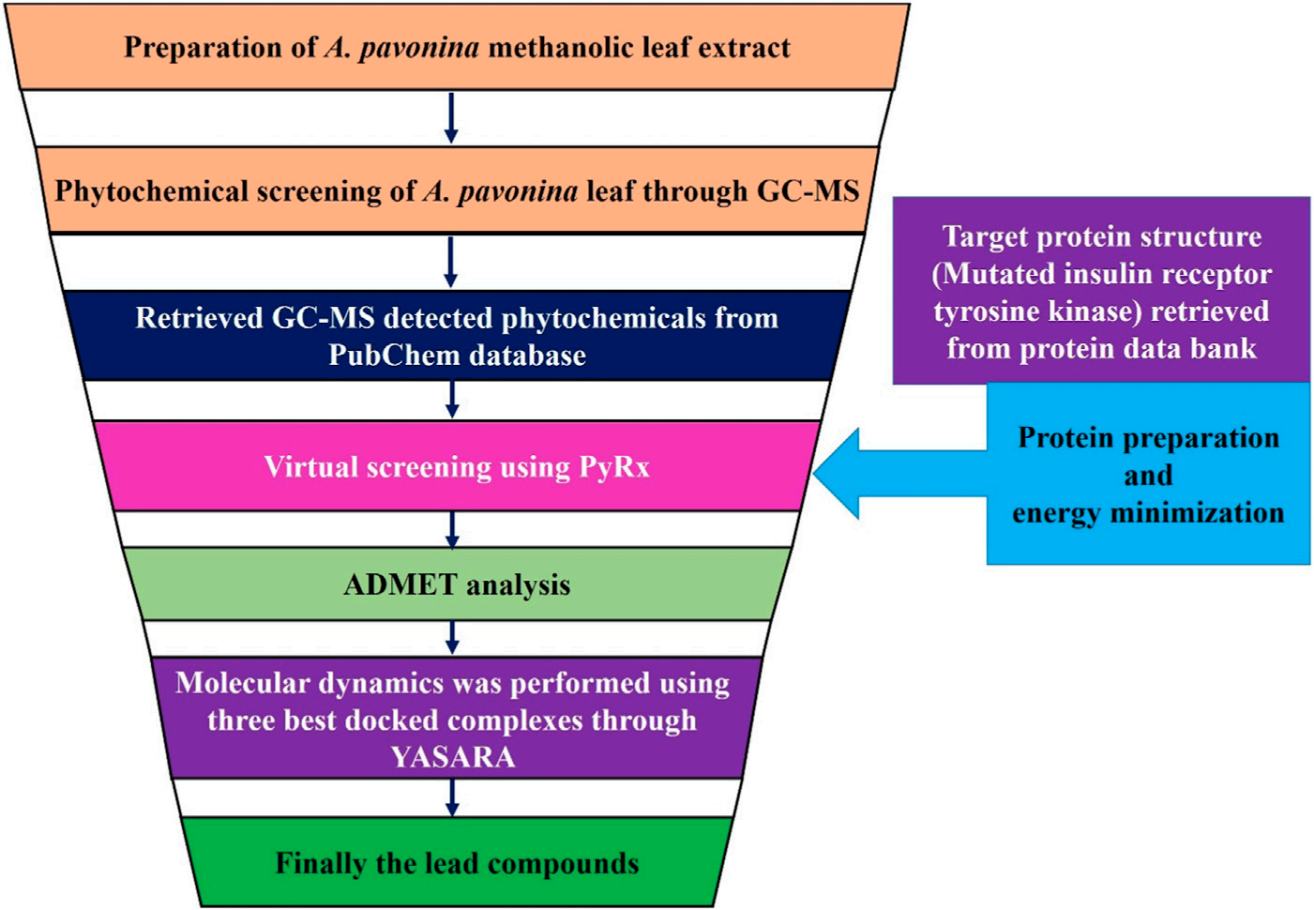
Overall workflow to find out the potential phytochemicals.

The overall workflow for identifying the potential activators of 5hhw protein is shown in Figure 1.

### 2.1 Leaf extract preparation

The leaves of *A. pavonina* were first washed with distilled water to remove dust and then air-dried for 7 days in a well-ventilated area. Next, the dried leaves were ground into a fine powder. A 5 g powdered sample was extracted with 150 mL of 100% methanol solvent to produce the crude extract containing a wide range of bioactive compounds. The extract was filtered using Whatman No. 4 filter paper, and the solvent was completely evaporated using a rotary evaporator to produce a dense residue. The completely evaporated extract was then weighed and stored at 4°C.

### 2.2 GC-MS analysis

The phytochemicals present in the methanolic leaf extract of *A. pavonina* were detected through GC-MS (Shimadzu, Japan; Model GC–MS QP 2020) analysis with an Electronic Ionization (EI) detector. A fused silica capillary column (SH-Rxi-5Sil MS 30m (L) X 0.25 mm (ID), X 0.25 um (DF)) was used at a temperature of 80.0°C. The samples were taken in a fixed split mode at 220.00°C. The oven was preheated at 80.0°C for 2 min, then 150.0°C for 3 min, and finally 280.0°C for 5 min. The ionization voltage was adjusted to 70 eV and the electron multiplier was set to 450 V. The unknown spectra of GC-MS were then compared to the spectra of existing chemicals housed in the NIST08s, NIST08 and NIST14 libraries. The names of the compounds and their concentrations were determined at different retention times (Kanthal et al., 2014). GC-MS Post Run software was used for data analysis.

Throughout this study, we bridge the gap between chemical composition and antidiabetic activity, starting with the identification of compounds through GC-MS and proceeding to the assessment of their potential interactions with 5hhw by molecular docking.

### 2.3 Molecular docking study

#### 2.3.1 Preparation of ligands

In the preparation of ligands, the structures of phytochemicals detected through the GC-MS technique from the methanolic leaf extract were retrieved from the PubChem database (Choudhary et al., 2019) in SDF format. Afterward, the Avogadro software was used to optimize the geometry of the phytochemicals (Snyder and Kucukkal, 2021).

#### 2.3.2 Preparation of protein

The crystal structure of the mutated insulin receptor tyrosine kinase protein (PDB ID: 5hhw) was retrieved from the Protein Data Bank (Stauffer et al., 2016) in PDB format. Heteroatoms were then removed using Discovery Studio software, and the cleaned protein was minimized and its energy optimized using Swiss PDB viewer software (Jin et al., 2019).

#### 2.3.3 Molecular docking

To investigate the interactions between the 5hhw protein and chemical compounds from *A. pavonina*, a molecular docking study was performed using the PyRx software with AutoDock Wizard (Dallakyan and Olson, 2015). The gasteiger charges for ligands CID: 104633 (0.002), CID: 536510 (−0.999), CID: 6432456 (−0.9997) and kollhman charges of protein (−8.31) were added and then converted to PDBQT format. The center and grid box size of the docked complexes were set at X: −14.5737 Å, Y: −15.6369 Å, Z: 18.2605 Å and X: 54.6511 Å, Y: 55.6782 Å, Z: 52.9565 Å, respectively, and all the amino acid residues of 5hhw protein were considered for blind docking. The final docking calculation was performed using PyRx, generating a total of nine poses for docking, and the best compounds were selected based on the highest binding energy. The binding interactions were analyzed using Discovery Studio software (Riyaphan et al., 2021).

### 2.4 ADMET analysis

Phytochemicals screened through molecular docking were analyzed for absorption, distribution, metabolism, excretion and toxicity (ADMET) to determine whether they possessed the anticipated features to be used as lead molecules. To analyze ADMET profiles and determine molecules’ correspondence to Lipinski’s rule of five, the pkcsm (Pires et al., 2015) and SwissADME (Daina et al., 2017) web servers were used.

### 2.5 Molecular dynamics

Molecular dynamics simulations were carried out using YASARA (Yet Another Scientific Artificial Reality Application) dynamics software and the AMBER (Assisted Model Building with Energy Refinement) 14 force field (He et al., 2020). Initial cleanup and optimization of the docked complexes were performed, as well as optimization of the hydrogen bond network. The topology files of ligands were auto-generated by General AMBER Force Field (GAFF) with AM1BCC charges. A cubic simulation cell was constructed using the TIP3P solvation model with a periodic boundary condition (Kadaoluwa Pathirannahalage et al., 2021). The distance between the cubic box and the edge of the protein was set to 12.5 Å, and the dimensions of the cubic box were 80 × 80 × 80 Å. The simulated system was neutralized at a pH of 7.4, 310 K, and 0.9% NaCl, representing physiological conditions (Kr et al., 2015). The particle mesh Ewald (PME) method was used to determine long-range electrostatic interactions with an 8.0 Å cutoff radius (Simmonett and Brooks, 2021). A time step of 2.0 fs was chosen for the simulation. The primary energy minimization was carried out using steepest gradient algorithms with simulated annealing techniques (5,000 cycles). The RMSD, Rg, SASA, hydrogen bonding and MMPBSA binding free energy were evaluated using the simulated trajectories (Baildya et al., 2021; Islam et al., 2021). The simulation was conducted for 100 ns, and the simulation trajectories were saved after every 100 ps (Patil et al., 2021).

## 3 Results and discussion

### 3.1 GC-MS analysis

**FIGURE 2:**
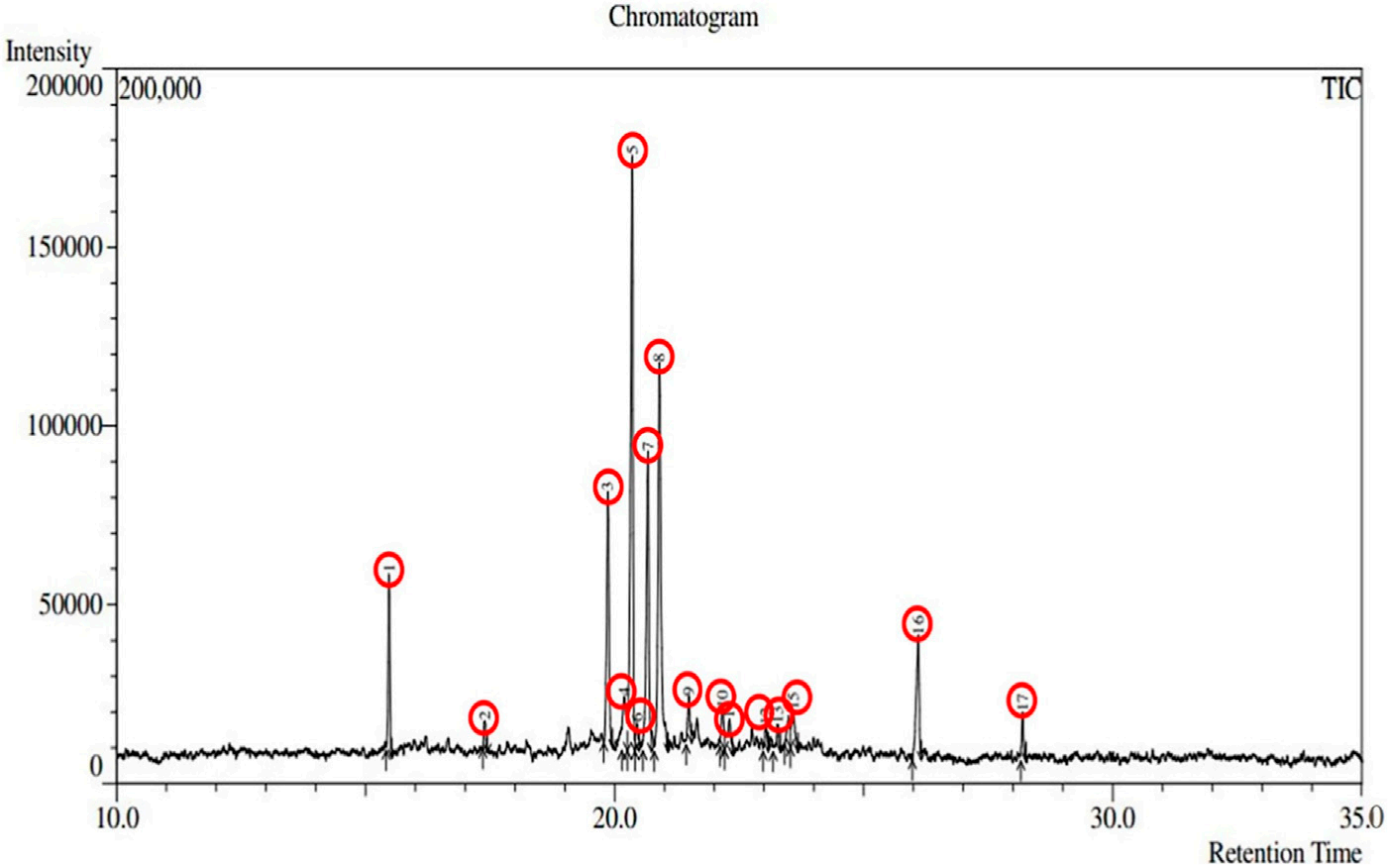
Chromatography of the chemical compounds found in methanolic leaf extract of *Adenanthera pavonina* by GC-MS analysis.

Plant-derived bioactive compounds have the potential to lower the complications associated with diabetes (Tran et al., 2020), and IR is a promising target for designing drugs for the curative treatment of diabetes mellitus. In this study, we identified a total of 17 phytochemicals present in *A. pavonina* leaf extract through GC-MS analysis (Figure 2; Table 1). Compound 3,7-Cyclodecadiene-1-methanol, .alpha.,.alpha.,4,8-tetramethyl was found to be the most abundant (29.15%) in this extract, followed by 2-(4-Ethenyl-4-methyl-3-prop-1-en-2-ylcyclohexyl)propan-2-ol (18.29%) and 2-Naphthalenemethanol, decahydro-.alpha.,alpha.,4a-trimethyl-8-methylene-, [2R-(2.alpha.,4a.alpha.,8a.beta.)] (14.15%) (Table 1). On the other hand, sabinene (27.9%) was the major compound of *A. pavonina* leaf oil, followed by D-limonene (14.79%), as detected through GC-MS analysis (El-Shazly et al., 2020). Although there was a significant difference in the concentrations of the compounds, some of the compounds were present at very low concentrations (<1%) in this leaf extract (Table 1).

**TABLE 1:** Quantitative result for methanolic leaf extract of *Adenanthera pavonina*.

ID	Name	RT	Area	m/z	Con.(%)
1	1H-3a,7-Methanoazulene, octahydro-1,4,9,9-tetramethyl	15.47	8,980	55.00	2.99
2	2,4,4,7a-Tetramethyl-1-(3-oxobutyl)octahydro-1H-indene-2-carboxylic acid	17.38	2076	82.00	0.69
3	5-Azulenemethanol, 1,2,3,3a,4,5,6,7-octahydro-.alpha.,.alpha.,3,8-tetramethyl-, [3S-(3.alpha.,3a.beta.,5.alpha.)]	19.86	33,445	59.00	11.16
4	Cyclohexanemethanol, 4-ethenyl-.alpha.,.alpha.,4-trimethyl-3-(1-methylethenyl)-, [1R-(1.alpha.,3.alpha.,4.beta.)]	20.19	5,617	59.00	1.87
5	3,7-Cyclodecadiene-1-methanol, .alpha.,.alpha.,4,8-tetramethyl	20.35	87,313	59.00	29.15
6	Terpineol	20.44	2,555	59.00	0.85
7	2-Naphthalenemethanol, decahydro-.alpha.,.alpha.,4a-trimethyl-8-methylene-, [2R-(2.alpha.,4a.alpha.,8a.beta.)]	20.67	42,382	59.00	14.15
8	2-(4-Ethenyl-4-methyl-3-prop-1-en-2-ylcyclohexyl)propan-2-ol	20.89	54,788	59.00	18.29
9	Benzenepropanol, .alpha.,.alpha.-dimethyl-	21.49	11,013	59.00	3.67
10	Diazoprogesterone	22.16	3,300	55.00	1.11
11	Pregnenolone carbonitrile	22.30	2,385	91.00	0.79
12	Cyclopentaneundecanoic acid, methyl ester	23.03	3,167	74.00	1.05
13	Oxacyclotetradeca-4,11-diyne	23.28	1952	91.00	0.65
14	Cyclopropane, 1-(2-methylene-3-butenyl)-1-(1-methylenepropyl)-	23.58	3,575	91.00	1.19
15	Caffeine	26.09	29,497	67.00	9.84
16	Cyclopentaneundecanoic acid, methyl ester	28.19	7,444	74.00	2.48
17	Picolinyl 7-tetradecenoate	16.02	3,100	62.00	0.58

RT , retention time, Con.(%) = Concentration (%).

### 3.2 Molecular docking

**FIGURE 3:**
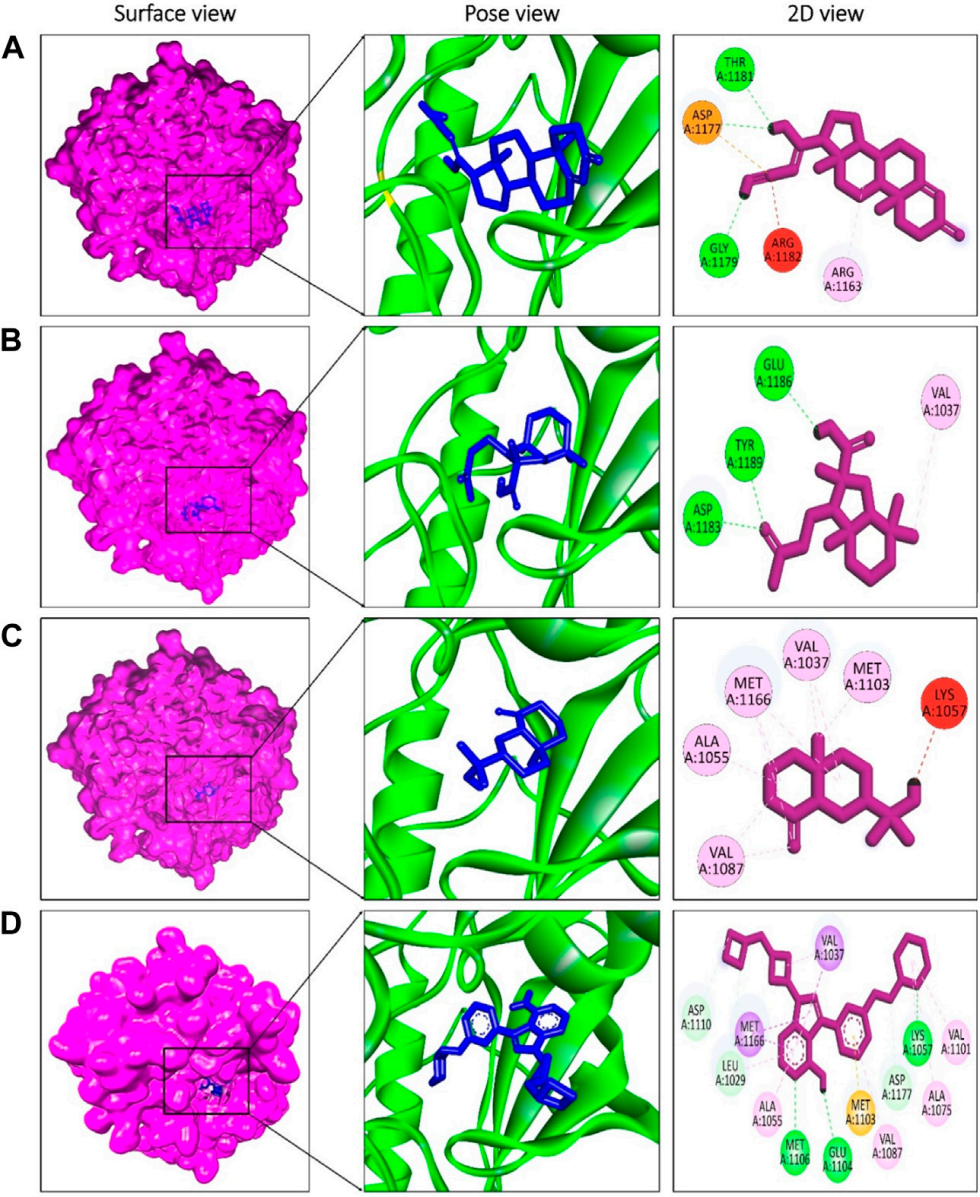
Molecular docking between 5hhw protein and compounds from *Adenanthera pavonina*; and surface, pose and 2D view of complexes 5hhw + CID: 104633 **(A)**, 5hhw + CID: 536510 **(B)**, 5hhw + CID: 6432456 **(C)** and reference molecule bound complex 5hhw + CID: 67035535 **(D)** respectively.

The presence of a mutation in the 5hhw domain of the IR prevents glucose uptake and metabolism, resulting in DMT2 (Cignarelli et al., 2019; Zhang et al., 2022). In our current study, we evaluated the *A. pavonina* leaf extract-derived bioactive compounds through *in silico* approach by targeting 5hhw protein to find out potential therapeutic drug candidates for DMT2. Based on their binding energy and number of interacting amino acid with the target protein 5hhw we have selected 3 compounds from 17 compounds for further *in silico* analysis (Supplementary Table S1). We used cis-(R)-7-(3-(azetidin-1-ylmethyl)cyclobutyl)-5-(3-((tetrahydro-2H-pyran-2-yl)methoxy)phenyl)-7H-pyrrolo[2,3-d]pyrimidin-4-amine (CID: 67035535) as a reference ligand because it was complexed with the crystal structure of the insulin receptor kinase domain. In the docking study, the binding energy for compound diazoprogesterone (CID: 104633) was −9.2 kcal/mol compared to the reference ligand CID: 67035535 (−10.5 kcal/mol). This was followed by the compounds 2,4,4,7a-Tetramethyl-1-(3-oxobutyl)octahydro-1H-indene-2-carboxylic acid (CID: 536510) and 2-Naphthalenemethanol, decahydro-.alpha.,.alpha.,4a-trimethyl-8-methylene-, [2R-(2.alpha.,4a.alpha., 8a.beta.)] (CID: 6432456), which exhibited binding energies of −6.9 kcal/mol and −6.6 kcal/mol, respectively (Table 2). The complex 5hhw + CID: 104633 exhibited two hydrogen bonds at A:THR1181 (2.88 Å) and A:GLY1179 (2.21 Å) (Figure 3A; Table 2). Similarly, the complex 5hhw + CID: 536510 was stabilized by three hydrogen bonds at A:GLU1186 (2.17 Å), A:TYR1189 (1.99 Å), and A:ASP1183 (2.27 Å) (Figure 3B; Table 2). However, the interaction between 5hhw and compound CID: 6432456 did not reveal any hydrogen bond but rather five alkyl bonds in the A chain of the 5hhw protein (Figure 3C; Table 2). In contrast, the interaction between 5hhw and reference ligand (CID: 67035535) exhibited three hydrogen bonds at A:LYS1057 (2.20 Å), A:GLU1104 (2.48 Å), and A:MET1106 (2.59 Å). The binding of these compounds to 5hhw may activate the IR by auto-phosphorylation and phosphorylation of intracellular substrates, thus initiating the insulin pathway and facilitating glucose uptake by the cell.

**TABLE 2:** Docking results of three phytochemicals of *Adenanthera pavonina* which showed the highest binding energy with 5hhw protein.

Complexes	Binding energy (kcal/mol)	Amino acid residues	Bond types	Distances (Å)
5hhw + CID: 104633	−9.2	A:THR1181	H	2.88
		A:GLY1179	H	2.21
		A:ASP1177	AC	2.40
		A:ARG1182	UPP	3.84
		A:ARG1163	A	4.54
5hhw + CID: 536510	−6.9	A:GLU1186	H	2.17
		A:TYR1189	H	1.99
		A:ASP1183	H	2.27
		A:VAL1037	A	4.29
5hhw + CID: 6432456	−6.6	A:VAL1087	A	3.88
		A:ALA1055	A	4.69
		A:MET1166	A	4.22
		A:VAL1037	A	3.59
		A:MET1103	A	4.70
		A:LYS1057	UDD	2.62
5hhw + CID: 67035535 (References ligand)	−10.5	A:LYS1057	H	2.20
		A:GLU1104	H	2.48
		A:MET1106	H	2.59
		A:LEU1029	CH	3.58
		A:ASP1110	CH	3.78
		A:ASP1177	CH	3.37
		A:ALA1055	A	5.23
		A:ALA1075	A	5.37
		A:VAL1087	A	5.25
		A:VAL1101	A	4.74
		A:VAL1037	PiS	4.88
		A:MET1166	PiS	3.93
		A:MET:1103	PS	3.57

H = hydrogen bond, CH, carbon hydrogen bond; AC, attractive charge; UPP, Unfavorable positive-positive bond, A = alkyl bond, UDD, Unfavorable donor-donor bond, PiS = Pi-Sigma, PS, Pi-Sulfur bond.

An *in silico* study has found that plant-derived polyphenols, such as flavonoids, can trigger the activation loop of IR, suggesting that they can be considered activators of IR (Lim et al., 2021). However, some *in vitro* and *in vivo* studies suggest that *A. pavonina*-derived phytochemicals possess antidiabetic activity (Wickramaratne et al., 2016; Vieira et al., 2018). The phytochemical galactomannans, isolated from *A. pavonina*, can reduce glycemia, total cholesterol, and triacylglycerol levels in streptozotocin-induced diabetic mice (Vieira et al., 2018). The methanolic leaf extract of *A. pavonina* significantly inhibits the alpha-amylase activity (IC_50_ value 16.16 ± 2.23 µg/mL) (Wickramaratne et al., 2016). It has been found that the seed aqueous extract of *A. pavonina* can reduce the development of diabetic nephropathy in streptozotocin-induced diabetic mice (Pandhare et al., 2012).

### 3.3 ADMET analysis

The compounds screened through molecular docking were chosen for ADMET analysis to evaluate their biocompatibility, pharmacokinetics, and toxicity, and to identify the lead compound. According to Lipinski’s rule, a lead compound should have certain properties, such as molecular weight ≤500 da, hydrogen bond acceptor ≤10, hydrogen bond donor ≤5 and LogP value ≤ 5. At least two violations are considered according to this rule (Abbasi et al., 2019). In the present study, it was found that all compounds met Lipinski’s rule (No violation). The bioavailability score of a compound determines its physiological activity (Jia et al., 2020). All three compounds were found to be physiologically active (>0.00), and their bioavailability scores were the same (0.55) (Table 3). The water solubility profile showed that compound CID: 536510 (−2.543) was water soluble, while compounds CID: 104633 (−4.101) and CID: 6432456 (−4.9) were moderately soluble according to the solubility scale, which is insoluble < −10 < poorly < −6 < moderately < −4 < soluble < −2 < very <0 < highly (Wang et al., 2021). The blood-brain barrier (BBB) permeability of a drug is crucial for its ability to reach the brain (Daina and Zoete, 2016). The BBB and CNS permeability of compounds CID: 104633, CID: 536510, and CID: 6432456 were 0.084 and −1.843, 0.361 and −1.104, and 0.634 and −1.858, respectively. Furthermore, all screened compounds showed positive (No) results in the human ether-a-go-go (hERG) I inhibitor test, and no toxicity was found in terms of the AMES test, however, compound CID: 536510 was found as hepatotoxic (Table 3).

**TABLE 3:** Pharmacological and toxicity predictions of the selected compounds using pkCSM and SwissADME tools where every compound had almost favorable drug-likeness properties.

Parameters	CID: 104633	CID: 536510	CID: 6432456
Molecular weight	340.46	260.42	222.308
Molecular formula	C_21_H_28_N_2_O_2_	C_17_H_28_N_2_	C_15_H_26_O
Hydrogen bond donor	0	1	1
Hydrogen bond acceptor	4	2	1
Rotatable bonds	2	4	1
LogP	4.0042	2.9244	3.92
Surface Area	149.230	117.540	99.941
Lipinski	Yes; 0 violation	Yes; 0 violation	Yes; 0 violation
Bioavailability score	0.55	0.55	0.55
Water solubility	−4.101	−2.543	−4.9
Human intestinal absorption	97.088	92.973	94.296
Blood brain barrier	0.084	0.361	0.634
CNS permeability	−1.843	−1.104	−1.858
P-Glycoprotein 1 inhibitor	Yes	No	No
CaCo2 Permeability	0.662	1.497	1.508
CYP2D6 substrate	No	No	No
Oral Rat Acute Toxicity (LD50)	2.717	2.903	1.727
AMES Toxicity	No	No	No
Hepatotoxicity	No	Yes	No
hERG I Inhibitor	No	No	No

### 3.4 Molecular dynamics

Molecular dynamics simulations were conducted to assess the stability of the hit complexes during the 100 ns simulation period. The RMSD value was utilized as the standard measure of structural distance between coordinates (Velázquez-Libera et al., 2020). The three best hit compounds were selected for molecular dynamics simulations to analyze their stability and identify potential inhibitors in a time-dependent manner for 100 ns Five parameters, including the root mean square deviation (RMSD), radius of gyration (Rg), solvent-accessible surface area (SASA), hydrogen bonds and MMPBSA binding free energy of the hit complexes were investigated.

**FIGURE 4:**
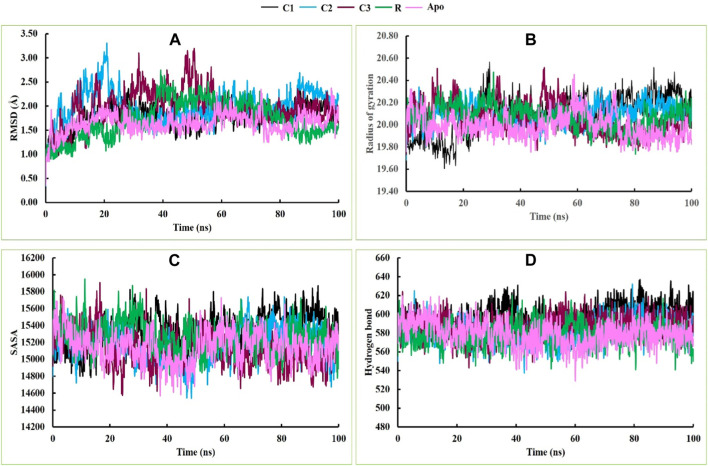
The molecular dynamics simulations of the docked C1, C2, C3, R and Apo complexes, where **(A)**, **(B)**, **(C)** and **(D)** indicate RMSD, Rg, SASA and hydrogen bonds respectively.

In this study, all four complexes demonstrated satisfactory RMSD, although there was some fluctuation in the data at the very beginning of the simulation period (Figure 4A). The initial RMSD values of the four complexes were below 1 Å, but after a few ns, the RMSD values of all complexes increased. Complex C2 (5hhw + CID: 536510) exhibited more fluctuation than complexes C1 (5hhw + CID: 104633), C3 (5hhw + CID: 6432456), reference ligand-bound complex R (5hhw + CID: 67035535), and Apo protein after 20 ns. This might be due to the conformational variability of the C2 complex (Saha et al., 2023). After this period, both C1 and C2 maintained a stable RMSD value for the rest of the simulation period, whereas R and Apo maintained stability with C3 until 80 ns and then R showed a downward trend with a slight increase at the end. However, the C3 complex exhibited an increased RMSD value until 60 ns, and then finally followed the stable trend with the other complexes (Figure 4A).

During the 20 ns of the simulation period, the Rg values of C2, C3, R and Apo protein showed an increasing trend, while C1 exhibited a decreasing movement. From 20 ns to 75 ns, all complexes demonstrated a stable profile. Afterward, C1 and C2 showed an upward movement, while C3, R and Apo showed a downward trend. However, at the end of the simulation period, they converged at nearly the same point (Figure 4B). The Rg profiles of C3, R, and Apo maintained almost the same trend, while C1 and C2 exhibited slight fluctuations during the simulation period due to the folding and changes in the conformation of the complexes (Rodrigues et al., 2018). The SASA profiles of C1, C2, C3, R and Apo were satisfactory, and they exhibited similar trends and stable profiles throughout the entire simulation period (Figure 4C). Similarly, the hydrogen bonding in these complexes also followed the same trend as the SASA profile throughout the simulation time (Figure 4D).

**FIGURE 5:**
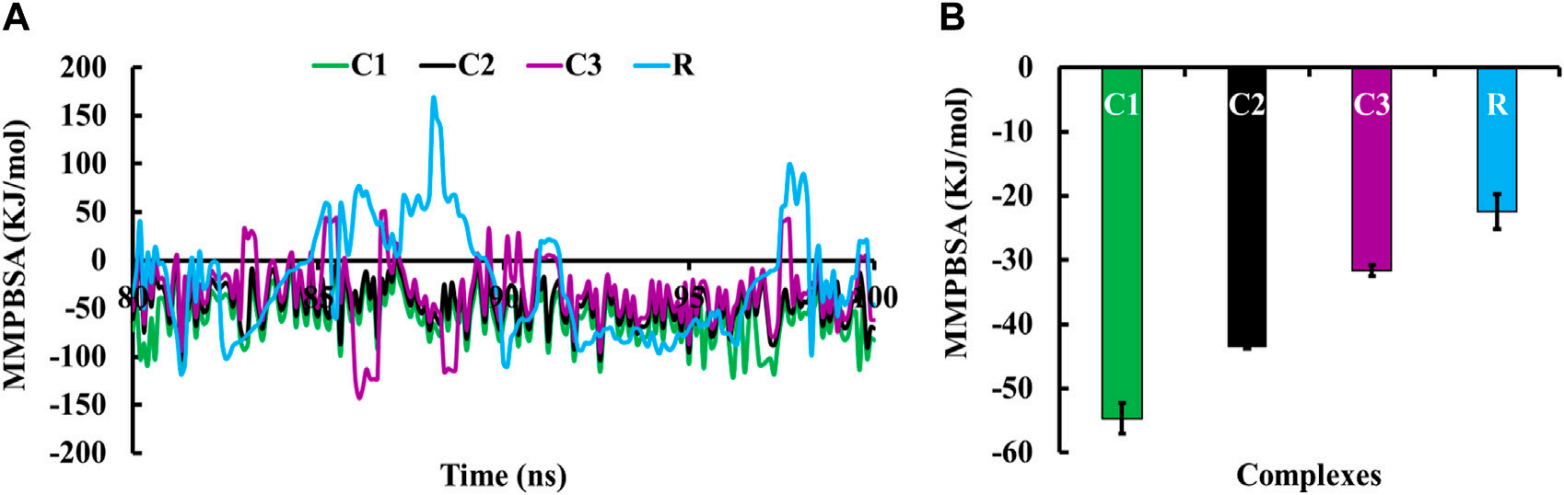
MMPBSA binding free energies of complexes C1, C2, C3 and R; where **(A)** displays the MMPBSA binding free energy at the 20 ns simulation period, and **(B)** shows the average MMPBSA binding free energy.

Molecular Mechanics/Poisson-Boltzmann Surface Area (MMPBSA) was used to calculate the binding free energy of protein-ligand interactions (Lyndem et al., 2023). The MMPBSA binding free energy of all complexes during the last 20 ns simulation period is shown in Figure 5A. The average MMPBSA binding free energies of complexes C1, C2, C3, and R were −54.69 ± 2.40, −43.53 ± 0.28, −31.64 ± 0.88 and −22.40 ± 2.72 kJ/mol, respectively (Figure 5B). The negative MMPBSA binding free energy indicates the stability of the C1, C2, C3 and R complexes (Figure 5A).

**FIGURE 6:**
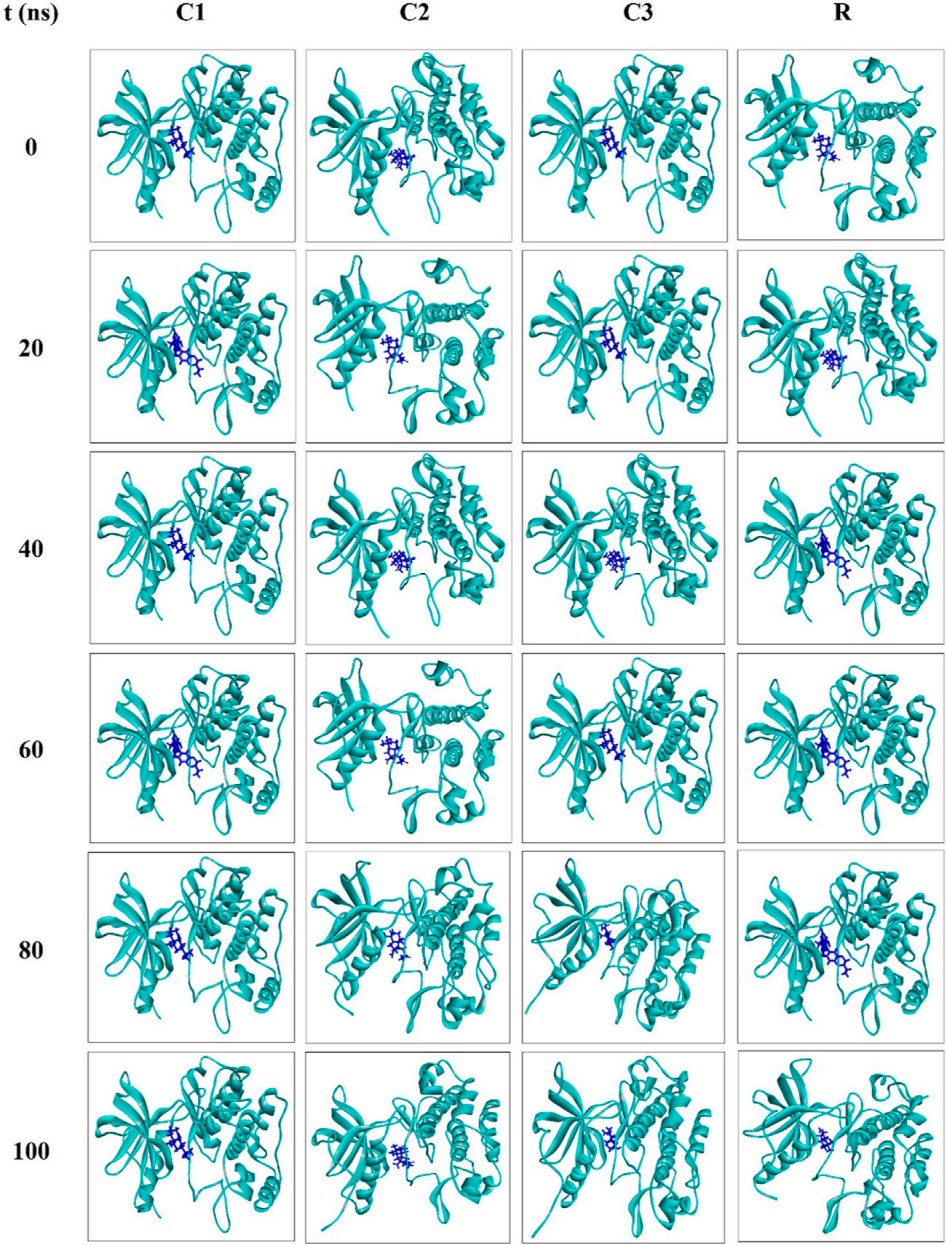
Snapshots of the molecular dynamics simulations for the complexes C1, C2, C3 and R at 0, 20, 40, 60, 80 and 100 ns?

Snapshots of molecular dynamics simulations were extracted at 0, 20, 40, 60, 80 and 100 ns to confirm the binding of ligands to the receptor. The findings indicate that the ligands remained bound to the receptor throughout the simulation period (Figure 6). Overall, the results of the molecular dynamics simulations suggest that CID: 104633, CID: 536510 and CID: 6432456 could serve as lead compounds.

### 3.5 Protein-ligand interactions after simulation

**FIGURE 7:**
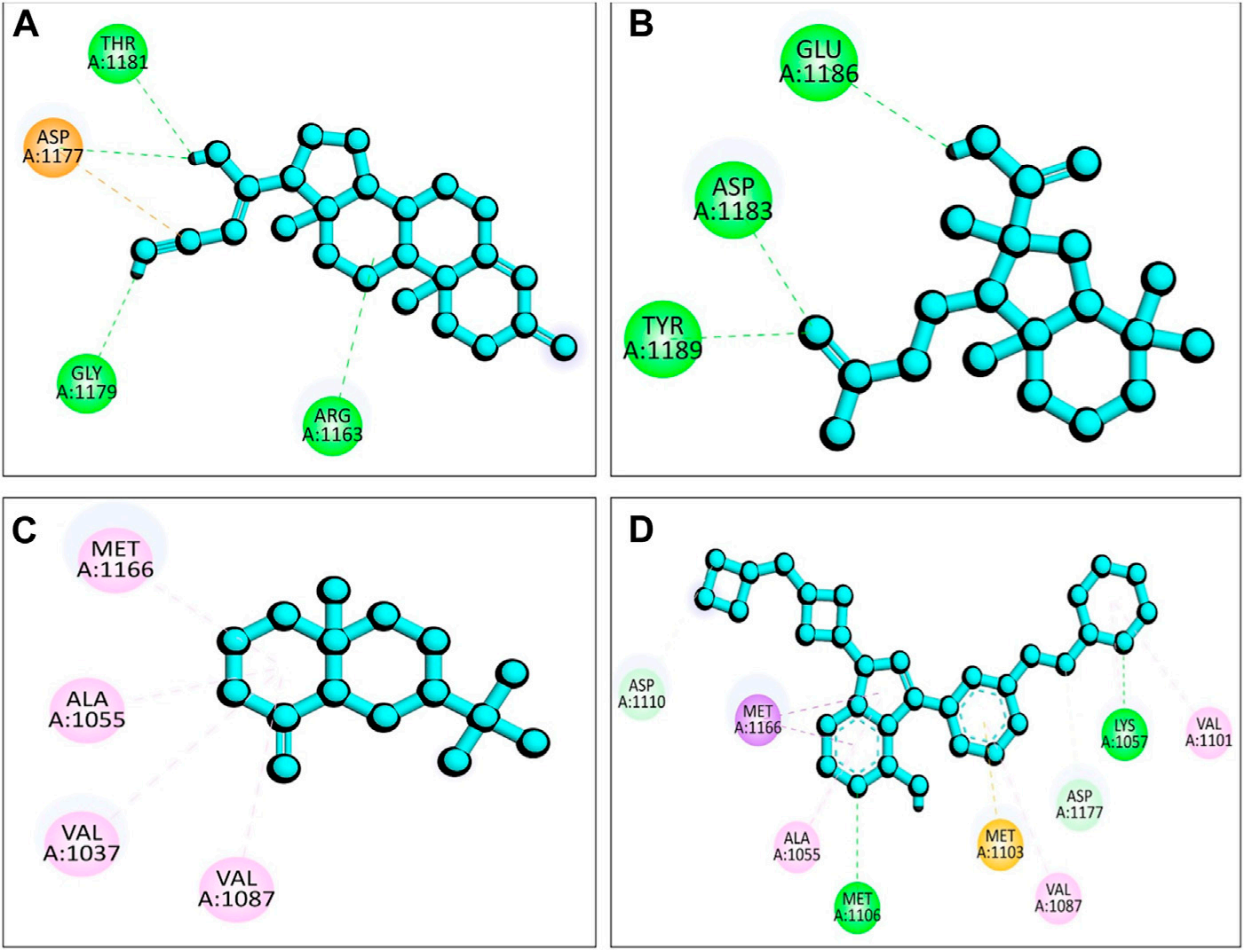
The 2D view of protein-ligand interactions after 100 ns simulation period where **(A)** 5hhw + CID: 104633, **(B)** 5hhw + CID: 536510, **(C)** 5hhw + CID: 6432456 and **(D)** 5hhw + CID: 67035535.

In Figure 7, the interaction between the protein and ligand after a 100 ns simulation period is depicted (Saha and Nath Jha, 2023). The results revealed that the number of interactions between the complexes 5hhw + CID: 104633, 5hhw + CID: 536510, 5hhw + CID: 6432456 and 5hhw + CID: 67035535 was 4, 3, 4 and 9 respectively (Table 4), which closely resembled the docking interactions presented in Table 2.

**TABLE 4:** Interactions between protein and ligand after 100 ns simulation period.

Complexes	Amino acid residues	Bond types	Distances (Å)
5hhw + CID: 104633	A:ARG1163	H	4.54
	A:GLY1179	H	2.21
	A:THR1181	H	2.88
	A:ASP1177	AC	3.26
5hhw + CID: 536510	A:ASP1183	H	2.17
	A:GLU1186	H	2.25
	A:TYR1189	H	2.03
5hhw + CID: 6432456	A:VAL1037	A	5.37
	A:ALA1055	A	4.98
	A:VAL1087	A	5.12
	A:MET1166	A	4.20
5hhw + CID: 67035535 (References ligand)	A:LYS1057	H	2.20
	A:MET1106	H	2.59
	A:ASP1110	CH	3.78
	A:ASP1177	CH	3.37
	A:ALA1055	A	3.96
	A:VAL1087	A	5.25
	A:VAL1101	A	4.74
	A:MET1166	PiS	3.66
	A:MET:1103	PS	3.57

## 4 Conclusion

The phytochemicals Diazoprogesterone, 2,4,4,7a-Tetramethyl-1-(3-oxobutyl)octahydro-1H-indene-2-carboxylic acid, and 2-Naphthalenemethanol, decahydro-.alpha.,.alpha.,4a-trimethyl-8-methylene-, [2R-(2.alpha.,4a.alpha.,8a.beta.)], derived from *A. pavonina*, showed potential activity in molecular docking studies with the 5hhw protein however their activity is comparatively lower compared to the control, and exhibited positive results in ADMET analysis, indicating favorable pharmacokinetic and safety properties. Molecular dynamics simulations further supported their strong binding interactions with the 5hhw receptor over time. These findings suggest that these phytochemicals hold promise as potential activators of 5hhw protein, making them possible candidates for the development of novel therapies for diabetes mellitus. Nevertheless, to ascertain their therapeutic potential fully, additional *in vitro* and *in vivo* investigations are essential, followed by rigorous clinical trials to determine their safety, efficacy, and appropriate dosages for human use. The use of these phytochemicals as activators of 5hhw protein could represent a significant step towards finding new and effective treatments for diabetes mellitus, but comprehensive research and validation are critical before their clinical application.

## Data Availability

The raw data supporting the conclusion of this article will be made available by the authors, without undue reservation.
