# Supplementary material for: Evaluation of Adenanthera pavonina-derived compounds against diabetes mellitus: insight into the phytochemical analysis and in silico assays
Source: Front Mol Biosci. 2024 Mar 27;10:1278701. doi: 10.3389/fmolb.2023.1278701 (PMC11004346; doi:10.3389/fmolb.2023.1278701)
Supplement: Supplementary file 1 [file DataSheet1.PDF]

Supplementary file

***Adenanthera pavonina*-derived compounds to identify potential activators of mutated insulin receptor tyrosine kinase from diabetes mellitus: insight into the phytochemical analysis and *in silico* assays**

Table S1. List of compounds, their binding energy and interacting amino acid residue with the target protein 5hhw.

Compounds name	PubChem CID	Interacting amino acid	Binding energy (kcal/mol)
1H-3a,7-Methanoazulene, octahydro-1,4,9,9-tetramethyl	29408	1	-4.1
(1R,2R,8aS)-2,4,4,7a-Tetramethyl-1-(3-oxobutyl)-trans-hydrindan-2-carboxylic acid	536510	4	-6.9
5-Azulenemethanol, 1,2,3,3a,4,5,6,7-octahydro-.alpha.,.alpha.,3,8-tetramethyl-, [3S-(3.alpha.,3a.beta.,5.alpha.)]	6432250	4	-6.2
Cyclohexanemethanol, 4-ethenyl-.alpha.,.alpha.,4-trimethyl-3-(1-methylethenyl)-, [1R-(1.alpha.,3.alpha.,4.beta.)]	11064030	3	-6.0
3,7-Cyclodecadiene-1-methanol, .alpha.,.alpha.,4,8-tetramethyl	6432240	3	-5.7
Terpineol	17100	4	-6.4
2-Naphthalenemethanol, decahydro-.alpha.,.alpha.,4a-trimethyl-8-methylene-, [2R-(2.alpha.,4a.alpha.,8a.beta.)]	6432456	6	-6.6
Cyclohexanemethanol, 4-ethenyl-.alpha.,.alpha.,4-trimethyl-3-(1-methylethenyl)-, [1R-(1.alpha.,3.alpha.,4.beta.)]	11064030	3	-4.8
Benzenepropanol, .alpha.,.alpha.-dimethyl-	7632	4	-.6.5
Diazoprogesterone	104633	5	-9.2
Pregnenolone carbonitrile	15032	3	-6.0
Cyclopentaneundecanoic acid, methyl ester	535041	4	-6.3
Oxacyclotetradeca-4,11-diyne	560917	3	-5.2
Cyclopropane, 1-(2-methylene-3-butenyl)-1-(1-methylenepropyl)-	562016	4	-6.4
Caffeine	2519	3	-4.9
Cyclopentaneundecanoic acid, methyl ester	535041	4	-6.4
Picolinyl 7-tetradecenoate	91700058	4	-5.9
Control	67035535	13	-10.5
